# Changes in youth public psychiatric service utilization during the COVID-19 pandemic in Italy: an observational study

**DOI:** 10.3389/fphar.2026.1708009

**Published:** 2026-02-09

**Authors:** Ippazio Cosimo Antonazzo, Carla Fornari, Manuel Zamparini, Giacomo Crotti, Alberto Zucchi, Pietro Ferrara, Alexandra Maria Piraino, Paolo Angelo Cortesi, Lorenzo Losa, Giampiero Mazzaglia, Lorenzo Giovanni Mantovani

**Affiliations:** 1 Research Centre on Public Health, University of Milano-Bicocca, Monza, Italy; 2 IRCCS, Istituto Auxologico Italiano, Milan, Italy; 3 Health Protection Agency of Bergamo (ATS Bergamo), Bergamo, Italy; 4 Department of Environmental and Prevention Sciences, University of Ferrara, Ferrara, Italy

**Keywords:** antidepressants, COVID-19, emergency department, Italy, time-series analysis

## Abstract

**Introduction:**

Social restrictions due to the COVID-19 pandemic have impacted the lives of children and adolescents worldwide. In this study, we aim to evaluate the impact of lockdown periods implemented in Italy during the SARS-CoV-2 waves on the mental health of minors.

**Methods:**

A population-based study using Italian healthcare administrative databases was conducted. Weekly time series of emergency department (ED) accesses/hospitalizations for psychiatric disorders, along with the incidence of antidepressant/antipsychotic (AD/AP) drug users, were defined from 1 January 2017 to 31 December 2022. An interrupted time-series analysis was used to assess the effect of lockdowns on the observed outcomes. The results were reported as incidence rate ratios (IRRs) with their respective 95% confidence intervals (95% CIs).

**Results:**

The first lockdown was associated with an abrupt reduction in both the incidence of AD/AP use (IRR = 0.49; 95% CI = 0.34–0.72) and ED accesses/hospitalizations for psychiatric disorders (IRR = 0.15; 95% CI = 0.10–0.23) compared to the pre-pandemic period. The incidence of AD/AP medication users showed a marked increase in both the post-first lockdown period (IRR = 2.48; 95% CI = 1.71–3.59) and the post-second lockdown period (IRR = 1.26; 95% CI = 1.07–1.48). ED accesses/hospitalizations showed a marked increase in the post-first lockdown period (IRR = 1.90; 95% CI = 1.40–2.59).

**Conclusion:**

The different lockdowns implemented during the COVID-19 outbreaks markedly affected the mental health of minors, as suggested by the increased use of AD/AP medications, which can be considered a proxy of diseases. The increased use of AD/AP medications in the observed period calls for interventions to improve the mental health of the population. These findings highlight the need to ensure effective public health interventions to be prepared for future crises.

## Highlights

What is known
• The COVID-19 outbreak had a marked impact on the mental health of the population. Stressful events, such as the pandemic period, have been associated with an increased risk of developing anxiety disorders, depression, and post-traumatic stress disorder.
What is new
• Findings reveal that the pandemic period profoundly impacted the mental health of young individuals (children and adolescents). The results suggest an increased need for care and support for this population.
• The findings underscore the necessity of developing preventive strategies to ensure adequate mental healthcare for this young population, particularly during stressful events.

## Background

The COVID-19 outbreak led to unprecedented disruptions in education, employment, financial security, and social connectedness due to the implementation of public health measures aimed at reducing virus transmission (Kerr et al., 2021). The implementations of these measures, particularly during the early phases of the pandemic, were associated with an increase in psychiatric and neuropsychiatric disorders, including depression, anxiety, and cognitive deficits in both young and adult individuals (Taquet et al., 2021; Taquet et al., 2022; Bowe et al., 2023; Hamidi et al., 2024; Chen et al., 2025).

The COVID-19 outbreak had a profound impact on children and adolescents. Previous studies reported that pediatric populations who experienced enforced isolation or quarantine were five times more likely to require mental health services and exhibited higher levels of post-traumatic stress. In this population, stressful events such as lockdowns have also been associated with an increased risk of developing anxiety disorders, post-traumatic stress disorders, or major depressive disorders (Bai et al., 2022).

During the outbreak, studies initially reported a decrease in emergency department visits and psychiatric outpatient and inpatient service usage due to stricter public health restrictions and fear of virus transmission (Leff et al., 2021; Chadi et al., 2021; Finkelstein et al., 2021; Leeb et al., 2020; Díaz de Neira et al., 2021; Berger et al., 2022; Williams et al., 2021; Lazzerini et al., 2020; Mantica et al., 2020; Bortoletto et al., 2022). Conversely, some studies analyzing data from the first wave through the subsequent 6 months reported a marked increase in psychiatric-related healthcare service use compared to the pre-COVID-19 period, particularly among female adolescents. However, other studies observed a continued decline in psychiatric service utilization during the initial months of the COVID-19 outbreak (Reece and Sams, 2022; Penner et al., 2022). Similarly, during the early phases of the COVID-19 pandemic, there was an observed increase in the use of antidepressants, hypnotics, and anxiolytics among individuals aged 12–18 years (Levaillant et al., 2023; Couturas et al., 2024; Bliddal et al., 2023). Although different studies have investigated the short-term effects of lockdowns on adolescents, evidence on their long-term impact remains scarce. The analysis of long-term impacts of lockdowns may provide insight into potential rebound effects or other phenomena associated with the introduction and lifting of lockdown measures on the population’s mental health. For this reason, we aimed to investigate both the short- and long-term impacts of lockdown measures, implemented in one of the populations most affected by the COVID-19 pandemic, in young individuals.

## Methods

### Study design and data sources

This observational study was based on the healthcare administrative database (HAD) of the Italian National Health Service (Servizio Sanitario Nazionale, SSN, for simplicity referred to as the “NHS” in this article) for the province of Bergamo in Lombardy, which covers a population of more than 1 million inhabitants. Italy has a tax-funded universal NHS, organized into three levels: national, regional, and local. The HAD contains data on healthcare services reimbursed by the Italian NHS to all citizens. For this study, the following HADs were used: (i) the archive of demographic and administrative data on individuals living in the catchment area who receive national healthcare service assistance; (ii) the pharmacy claim registry, which provides information (i.e., dispensing date, substance name, and anatomical therapeutic chemical [ATC] code) on all community prescriptions reimbursed by the NHS; (iii) emergency department access, which provides information on date of access, date of discharge/hospitalization, and one main and five secondary diagnoses, coded using the International Classification of Diseases, Ninth Revision, Clinical Modification (ICD-9-CM); (iv) hospital discharge records (SDOs), which contain data on access and discharge, along with diagnosis information for all hospitalized individuals. We adopted an interrupted time-series design, a method previously described in other publications, aimed at assessing the impact of the first lockdown on the general population (Antonazzo et al., 2021a; Antonazzo et al., 2021b). The study was conducted in accordance with the Declaration of Helsinki. This study was approved by the “Comitato Etico Territoriale Lombardia 6” of the Lombardy Region, under study protocol 0053179/24 on 18 October 2024.

### Study population and outcomes

The study included a dynamic cohort of individuals aged <18 years receiving national healthcare service coverage under the jurisdiction of the Health Protection Agency (HPA) of Bergamo from 1 January 2017 to 31 December 2022.

To evaluate the use of psychiatric medications in the study population, a time series of subjects with at least one dispensing dose of antidepressant or antipsychotic (ATC: N06A and N05A) drugs during the study period was computed. Individuals were classified as incident users if no AD/AP dispensations were recorded in the year preceding the dispensing date. Then, the weekly incidence of psychiatric medication use was calculated as the number of incident users in the corresponding week divided by the number of inhabitants living in the catchment area of HPA-Bergamo as of 1 January of the corresponding calendar year.

The time series of ED accesses/hospitalizations due to psychiatric conditions included all individuals who accessed an ED or were hospitalized during the study period with a psychiatric diagnosis (ICD9-CM: 291, 292, 295, 296, 297, 298, 300–309, and 311). The weekly rate of ED accesses/hospitalizations was computed by dividing the number of cases occurring in the corresponding week by the number of inhabitants living in the HPA-Bergamo catchment area as of 1 January of the corresponding year.

### Statistical analysis

To examine the effect of different lockdown measures on study outcomes, the observation period was divided into five segments.

- Pre-first lockdown period from 1 January 2017 to 8 March 2020;
- First lockdown period from 9 March 2020 to 14 June 2020;
- Post-first lockdown period from 15 June 2020 to 6 November 2020.
- Second lockdown period from 7 November 2020 to 27 June 2021;
- Post-second lockdown period from 28 June 2021 to 31 December 2022 (Ministero della Salute, 2021).

Since the first COVID-19 cases were identified in Lombardy in February, the start date for the first lockdown for the analysis of ED accesses/hospitalizations due to psychiatric conditions was set to 24 February 2020 to account for the potential rapid decrease in the use of these services at the start of the emergency.

An interrupted time-series (ITS) approach (Wagner et al., 2002) was applied to estimate the effects of lockdown measures on the observed time-series outcomes. ITS is a quasi-experimental design used to evaluate the longitudinal effects of time-delimited interventions while accounting for seasonality and secular trends. Specifically, a quasi-Poisson-generalized additive model (Wagner et al., 2002) was employed, with the weekly count of the observed outcome serving as the response variable (Y) and the reference population acting as an offset variable to transform the count outcome into incidence.

The fitted model was expressed as follows:

Log[E(Y_i_)] = β_0_ + f(week_i_) + f(month_i_) + β_1_I(holiday_i_) + β_2_I(first lockdown_i_) + β_3_(first lockdown week_i_)+ β_4_ I(post-first lockdown_i_) + β_5_ (post-first lockdown week_i_) + β_6_ I(Second lockdown_i_) + β_7_ (second lockdown week_i_) + β_8_ I(post-second lockdown_i_) + β_9_ (post-second lockdown week_i_), with Yi as the weekly count of the study outcome (i is the time unit indicator), f(weeki) as a nonlinear function of the week (spline function), and a dummy holiday indicator (0 = no, 1 = yes) to account for time trends. Moreover, we included an indicator of the first lockdown period (0 = pre, 1 = from first lockdown) to estimate a possible level change (β_2_) in the time series after the implementation of the first lockdown measure (first lockdown week_i_) and a linear function of time during the first lockdown period to estimate a possible trend/slope change in the time series (β_3_) (Wagner et al., 2002). In the same way, we defined indicators and linear functions of time for the subsequent periods: post-first lockdown, second lockdown, and post-second lockdown.

For each intervention, the model estimated two parameters: a level change and a trend/slope change in the observed time series compared to the counterfactual trend estimated by the model in the absence of lockdown measure implementation. Specifically, in the model, a level change reflects the immediate impact of the intervention, whereas the change in trend/slope reflects a more gradual shift in the projected outcome over time (Wagner et al., 2002).

The statistical significance of the parameters and the goodness of fit of the model were used to choose the best model for each studied time-series analysis. We used a stepwise approach to identify the final model. A p-value ≤0.05 was considered statistically significant. The models were implemented separately for each study outcome. The ITS results were expressed as follows: parameter estimates and corresponding p-values for parameter significance and incidence ratios with 95% confidence intervals (95% CIs). The incidence rate ratio (IRR) was calculated as the exponential of the corresponding parameter. A sensitivity analysis was also performed considering the lag effect of lockdown measures for 4 or 3 weeks.

In this study, data processing and data analysis were performed using RStudio software (version 4.0.2, RStudio, PBC: Boston, MA, United States). In particular, ITS analysis was conducted using the “tsMODEL,” “splines,” and “mgcv” R packages.

## Results

### Incidence of psychiatric medication use

The analysis of AD/AP use showed a marked increase in the use of psychiatric drugs in the observed period. Specifically, the mean weekly incidence rate of psychiatric drug users among young individuals markedly decreased during the first lockdown period compared to the pre-lockdown period (2.23 per 100,000 inhabitants vs. 1.30 per 100,000 inhabitants). This was followed by a marked increase (2.79 per 100,000 inhabitants) during the post-first lockdown period compared with that during the first-lockdown period. This upward trend persisted throughout the subsequent periods. In particular, the post-second-lockdown period was characterized by a new marked increase in the incidence of psychiatric medication use (4.10 per 100,000 inhabitants) (Supplementary Table 1).

**FIGURE 1:**
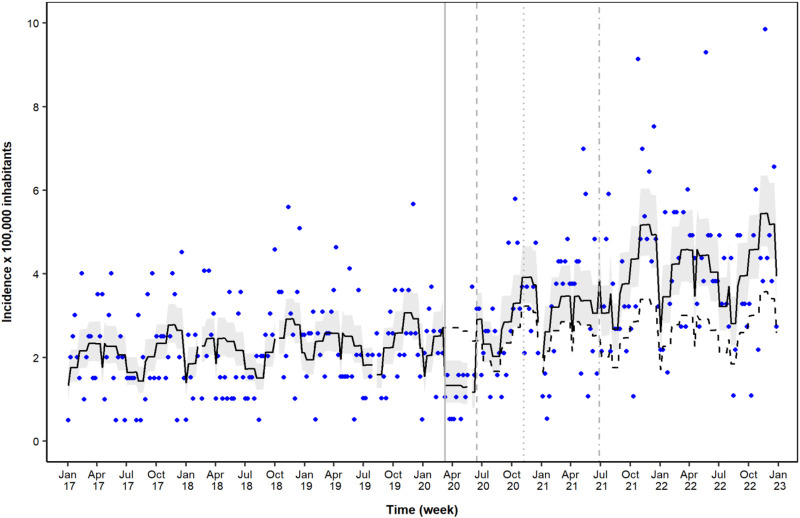
Time-series analysis of the incidence of psychiatric drug use during the study period. Legend: dots, raw data; solid black line, model prediction with 95% confidence intervals (gray bands); dashed black line, model counterfactual prediction; solid gray line, first lockdown period (8 March 2020); dashed gray line, post-first lockdown period (14 June 2020); dotted gray line, second lockdown period (6 November 2020); dash–dot gray line, post-second-lockdown period (27 June 2021).

As shown in the ITS analysis, during the first lockdown, an abrupt decrease in the incidence of new users of psychiatric medications was observed (IRR = 0.49; 95% CI = 0.34–0.72) (Table 1; Figure 1). Conversely, during the post-first lockdown period, a level change indicated a rapid increase in the incidence of psychiatric medication use (IRR = 2.48; 95% CI = 1.71–3.59). This increased incidence persisted during the following period, with an additional abrupt increase in the incidence of new users of psychiatric medications during the post-second lockdown period (IRR = 1.26; 95% CI = 1.06–1.48) (Table 1; Figure 1). In the sensitivity analysis, models including lag effects showed lower goodness of fit.

**TABLE 1:** Time-series analysis of the incidence of psychiatric drug use during the study period.

Model parameter	β	Incidence rate ratio	95% CI	p-value
First lockdown §	−0.714	0.490	0.335–0.717	≤0.001
Post-first lockdown §	0.907	2.477	1.708–3.590	≤0.001
Post-second lockdown §	0.228	1.256	1.064–1.483	≤0.01

The GAM model was also corrected for holiday, month, and the week’s spline function (K = 7); §, level change; *, slope change.

### Emergency department access and hospitalization due to psychiatric conditions

The analysis of ED accesses/hospitalizations due to psychiatric conditions showed a marked decrease in service utilization in the observed period. Specifically, weekly ED accesses/hospitalizations due to psychiatric diseases markedly decreased during the first lockdown period compared to the pre-lockdown period (4.05 per 100,000 inhabitants vs. 10.67 per 100,000 inhabitants) (Supplementary Table 2). Conversely, the weekly incidence of these events markedly increased during the post-first lockdown period (8.03 per 100,000 inhabitants). Although not statistically significant, the subsequent period showed a slight increase in the study events (8.64 per 100,000 inhabitants).

**FIGURE 2:**
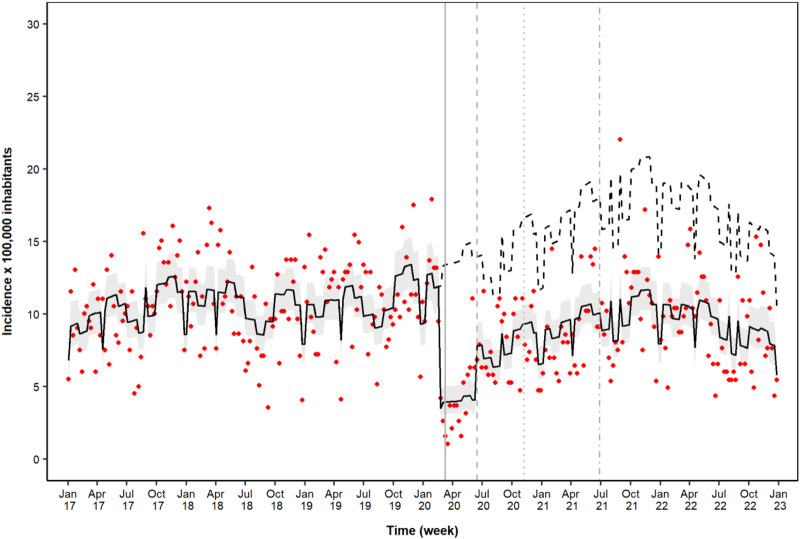
Time-series analysis of emergency department accesses/hospitalizations during the study period. Legend: dots, raw data; solid black line, model prediction with 95% confidence intervals (gray bands); dashed black line, model counterfactual prediction; solid line, first lockdown period (8 March 2020); dashed line, post-first lockdown period (14 June 2020); dotted line, second lockdown period (6 November 2020); dash–dot line, post-second-lockdown period (27 June 2021)

As observed in the ITS analysis, the implementation of the first lockdown was associated with an abrupt reduction in ED accesses/hospitalizations due to psychiatric diseases, as indicated by the level change in the model (IRR = 0.29; 95% CI = 0.22–0.39) (Table 2; Figure 2). This was followed by a mean level increase in the time series in the post-first lockdown period (IRR = 1.91; 95% CI = 1.40–2.60) (Table 2; Figure 2). In the sensitivity analysis, models including lag effects of up to 4 or 3 weeks confirmed the increasing trend after the initial level decrease during the first lockdown period, but showed lower goodness of fit.

**TABLE 2:** Time-series analysis of emergency department/hospitalization during the study period.

Model parameter	β	Incidence rate ratio	95% CI	p-value
First lockdown §	−1.225	0.294	0.220–0.392	≤0.001
Post-first lockdown §	0.645	1.906	1.402–2.593	≤0.001

The GAM model was also corrected for holiday, month, and the week’s spline function (K = 7); §, level change; *, slope change.

## Discussion

In this study, we provide insights into the impact of the COVID-19 pandemic and associated lockdowns on long-term psychiatric medication use and ED accesses/hospitalizations due to psychiatric diseases among young individuals in the Bergamo area. Bergamo, a densely populated province in Northern Italy, was one of the most severely affected areas during the initial wave of the pandemic. Using ITS analysis, we reveal the impact of the COVID-19 pandemic on young individuals (<18 years old) regarding psychiatric medication use and ED accesses/hospitalizations due to psychiatric diseases.

The findings indicate a substantial impact of the COVID-19 pandemic on young individuals. Specifically, during the first lockdown, the incidence of psychiatric medication use halved compared with that during the pre-lockdown period (−51%). Subsequently, the post-first lockdown period experienced a 2.5-fold increase in the incidence of psychiatric drug users in the study population. This incidence level persisted through the second lockdown and the post-second lockdown periods and was marked by an additional 25% increase in the incidence of the study phenomenon. Similar patterns in the incidence of psychiatric medication use were observed in other Italian studies (Di Valerio et al., 2024; Daneshmand et al., 2023; Antonazzo et al., 2022). For example, Di Valerio et al. reported a 50% reduction in the incidence of antidepressant use among young individuals (<19 years old) during the first lockdown period, followed by a 48% increase in subsequent periods (Di Valerio et al., 2024). Although these findings align with those of our study, direct comparisons are limited due to differences in study design. A Spanish study reported an immediate reduction in the prescription of anxiolytics, sedatives, hypnotics, and antidepressants among children and adolescents during the first lockdown period (Estrela et al., 2022). Additionally, a cohort study based on primary care data related to depression and anxiety among children and youth aged 10–25 years found an increased use of antidepressants and anxiolytics during the pandemic period (1 April 2020–31 December 2021) compared with the pre-pandemic period (Daneshmand et al., 2023). Although data on the impact of the COVID-19 pandemic on the incidence of psychiatric medication use in young individuals remain limited, several studies have highlighted the impact of lockdown measures on antidepressant and anxiolytic use in the general population (Antonazzo et al., 2022; Estrela et al., 2022). These findings suggest that the pandemic negatively affected both young and adult individuals during the lockdown and post-pandemic periods. In this regard, previous studies have reported an increase in psychiatric diseases in the youth population during the COVID-19 and post-COVID-19 periods (Kauhanen et al., 2023; Zhang-James et al., 2025; Cielo et al., 2021). Several stressors, including academic disruptions, widespread adoption of remote e-learning, economic hardship, social isolation, and disruptions to daily routines, emerged during the pandemic and significantly contributed to the deterioration of mental health in the young population (Kauhanen et al., 2023; Zhang-James et al., 2025; Cielo et al., 2021). These factors may have increased the risk of depression and anxiety disorders, ultimately leading to an increase in the use of psychiatric medications.

The COVID-19 pandemic also markedly affected ED accesses/hospitalizations due to psychiatric conditions among young individuals. During the first lockdown period, a substantial reduction in ED accesses and hospitalizations for psychiatric diseases was observed in the study population (−71%). This initial decrease was followed by an increase after the post-first lockdown period (90%). Several studies have documented the negative impact of the first lockdown on ED access for psychiatric conditions. Specifically, a reduction in the number of ED accesses/hospitalizations was reported (Leff et al., 2021; Dror et al., 2022; Davico et al., 2021). In our study, it is interesting to note that after the first lockdown period, ED accesses/hospitalizations for psychiatric conditions remained below the levels observed in the pre-pandemic period. Given the persistent increase in antidepressant and antipsychotic use observed in our study during the post-first lockdown phase, it is plausible that the population became less reliant on hospital-based care for managing psychiatric conditions and instead turned to alternative healthcare resources. In this regard, recent studies have reported an increase in primary care visits for anxiety and depression during the pandemic and the subsequent periods compared with the pre-pandemic era (Daneshmand et al., 2023; Bittner Gould et al., 2022; Kuitunen et al., 2023).

Data on ED accesses/hospitalizations and psychiatric medication use after the first lockdown period suggest that the management of children and adolescents may have shifted outside the hospital setting. Specifically, it is plausible that after the first wave, mental health problems were managed in primary care or by child psychiatrists in an outpatient setting, with ED accesses/hospitalizations used only for severe cases. This shift could partially explain the increasing trend in AD/AP use observed during the post-first-lockdown and subsequent periods, which was not mirrored by a corresponding increase in hospitalization for psychiatric conditions. In addition, after the first restrictive lockdown, subsequent lockdown periods were characterized by more relaxed measures, allowing the population to resume normal routines. Therefore, it is plausible that returning to complex daily routines after a longer period of enforced cohabitation with family and social isolation may have contributed to triggering psychiatric symptoms and, consequently, increased psychiatric medication use in this population (Clavenna et al., 2024). In addition, a potential rebound effect cannot be completely excluded, and the challenges faced by youth during lockdown, such as social isolation and fear for the safety of friends and family, might have contributed to an increased risk of psychiatric symptoms in individuals (Wang et al., 2022).

In this study, we analyzed the impact of different lockdown phases on AD/AP drug use and ED accesses/hospitalizations due to psychiatric conditions in young individuals (<18 years old). In this regard, potential differences by age group and sex cannot be completely ruled out. In fact, previous studies have suggested that adolescent female individuals were more affected than male individuals (Clavenna et al., 2024; Otter et al., 2024; Tiger et al., 2023; Fassmer et al., 2025; Leith et al., 2022; Overhage et al., 2023; Ridout et al., 2021). Future studies should be conducted to assess whether these trends persist over longer follow-up periods. Furthermore, studies should be conducted to assess the severity of psychiatric conditions in patients who present to the ED or require hospitalization, along with the treatment patterns involving AD/AP in these individuals. Furthermore, public health interventions should aim to improve the early detection of mental health problems in youth with mental issues and improve patients’ engagement. Finally, future studies should evaluate the quality of life and assess whether youth who experienced mental health problems during the prior COVID-19 and post-COVID periods are at an increased risk of relapse in adulthood.

The strength of this study is that it is one of the few investigations examining the multifaceted impact of the COVID-19 pandemic on the mental health of young individuals. The Italian region of Lombardy registered the largest COVID-19 outbreak, and some of its provinces, including Bergamo, were among the hardest hit in terms of deaths during the epidemic period (Conti et al., 2020). Additionally, the province of Bergamo was one of the first areas in Italy to experience widespread SARS-CoV-2 transmission. For this reason, data from this population are essential for understanding the impact of the virus’s spread, as political decisions implemented during this period could not have been influenced by events occurring in other Italian regions (as there was no preparedness in the area). Furthermore, considering the tremendous impact of SARS-CoV-2 spread in this area, this province represents a valuable example for studying the impact of COVID-19 on the mental health of the population. It provides valuable insights into the short- and long-term responses of the population to SARS-CoV-2 waves and containment measures. In addition, in Italy, ADs and APs are reimbursed by the national healthcare system and can be dispensed by pharmacies to the general population only with a medical prescription, regardless of their socioeconomic status. This provides detailed information on the use of such medications in the general population, thereby enabling (pharmaco)epidemiological studies to be conducted.

This study has some limitations that should be acknowledged. First, data on benzodiazepines, which are commonly used to treat mental health symptoms, were not included in the analysis. This class of drugs is not reimbursed by the Italian NHS, and, therefore, benzodiazepine dispensing data cannot be retrieved from the Italian HAD. Second, the Italian pharmacy claim database does not include information on drug indication. Third, although the pharmacy claim database provides dispensing data, which is more informative than prescription data, it does not necessarily reflect the medication use in the population (i.e., it does not offer insight into whether patients actually consumed the dispensed medications). Fourth, the ED access and hospital discharge record databases included only diagnostic data but did not account for the severity of the conditions. Moreover, potential diagnostic miscoding due to overwhelmed health systems during the waves cannot be completely excluded. Furthermore, there may have been potential under-recording of psychiatric diagnoses; although the first lockdown was a measure that persisted for the entire period, the restrictions associated with the second lockdown varied during the period according to the spread of SARS-CoV-2 in the Lombardy region. This may partially explain the different trends between psychiatric medication estimates and ED/hospitalization estimates. Emergency department accesses and hospitalizations were grouped and analyzed together. This did not allow the assessment of potential differences in the impact of COVID-19 on emergency department accesses and hospitalizations. Additionally, due to the study design, it is not possible to establish a causal relationship between the observed outcomes and the COVID-19 outbreak, even though the changes in AD/AP use and ED accesses/hospitalizations during the lockdowns and subsequent periods are evident. Finally, in this study, we assessed the impact of the COVID-19 pandemic in a heavily affected Italian area; therefore, geographical differences in virus diffusion may limit the generalizability of our findings worldwide. Similarly, the varying lockdown measures implemented in different countries during the pandemic may have had different impacts on drug use and healthcare resource utilization.

## Conclusion

The incidence of AD/AP users increased rapidly after the beginning of the COVID-19 pandemic and continued to increase during the subsequent periods. In contrast, ED accesses/hospitalizations due to psychiatric conditions decreased during the first wave of COVID-19 and never returned to pre-lockdown levels. These findings indicate that the mental health of young individuals (children and adolescents) has been adversely affected by the pandemic, suggesting an increased need for care and support for this population. Furthermore, they indicate that additional stressful events, such as the implementation of lockdown measures, can exacerbate mental health challenges within the population. These data can guide stakeholders in developing targeted strategies to ensure adequate medical care for youth individuals who experienced the pandemic period and inform the implementation of appropriate interventions during future public health emergencies. Future research should investigate the very long-term impact of COVID-19, along with other stressful events, on this population.

## Data Availability

The data that support the findings of this study are available from HPA-Bergamo, but restrictions apply to the availability of these data, which were used under license for the current study and so are not publicly available. Data are, however, available from the authors upon reasonable request and with permission of HPA-Bergamo. Correspondence and requests for materials should be addressed to I.C.A. (ippazio.antonazzo@unimib.it) and C.F. (carla.fornari@unimib.it).
