# Supplementary material for: Changes in youth public psychiatric service utilization during the COVID-19 pandemic in Italy: an observational study
Source: Front Pharmacol. 2026 Feb 9;17:1708009. doi: 10.3389/fphar.2026.1708009 (PMC12964291; doi:10.3389/fphar.2026.1708009)
Supplement: Supplementary file 1 [file Table1.docx]

**Title: Changes in youth public psychiatric service utilization during the COVID-19 pandemic in Italy: an observational study.**

**Authors:** Ippazio Cosimo Antonazzo 1,2,3, Carla Fornari 1, Manuel Zamparini 1, Giacomo Crotti 3, Alberto Zucchi 3, Pietro Ferrara 1,2, Alexandra Maria Piraino 1, Paolo Angelo Cortesi 1,2, Lorenzo Losa 1, Giampiero Mazzaglia 1, Lorenzo Giovanni Mantovani 1,2

**Affiliation:**

1 Research Centre on Public Health, University of Milano-Bicocca, 20900 Monza, Italy

2 IRCCS, Istituto Auxologico Italiano, 20145 Milan, Italy

3 Health Protection Agency of Bergamo (ATS Bergamo), 24121 Bergamo, Italy

**Corresponding author**

Carla Fornari

Research Centre on Public Health

University of Milano-Bicocca

20900 Monza, Italy

**Supplementary materials**

**Table 1. Weekly count and incidence of psychiatric drugs use among young individuals.**

| Outcome | Period | | | | | Relative Change (%) | | | |
| --- | --- | --- | --- | --- | --- | --- | --- | --- | --- |
|  | **Pre-first lockdown** | **First lockdown** | **Post-first lockdown** | **Second lockdown** | **Post-second lockdown** | **First lockdown Vs Pre-first lockdown** | **Post-first lockdown Vs first lockdown** | **Second lockdown Vs Post-first lockdown** | **Post-second lockdown Vs second lockdown** |
| Weekly count | |  |  |  |  |  |  |  |  |
| Mean ± SD | 4.37 ± 2.11 | 2.46 ± 1.61 | 5.29 ± 2.33 | 6.06 ± 2.82 | 7.54 ± 3.19 | -43.65 * | 114.73 * | 14.7 | 24.44 |
| Median (Q1-Q3) | 4 (3-5) | 2 (1-3) | 5 (4-6) | 7 (4-8) | 7 (5-9) |  |  |  |  |
| Weekly incidence per 100,000 | | |  |  |  |  |  |  |  |
| Mean ± SD | 2.23 ± 1.08 | 1.3 ± 0.85 | 2.79 ± 1.23 | 3.24 ± 1.51 | 4.1 ± 1.73 | -41.76 * | 114.73 * | 16.44 | 26.44 * |
| Median (Q1-Q3) | 2.06 (1.51-2.64) | 1.05 (0.53-1.58) | 2.64 (2.11-3.16) | 3.69 (2.11-4.3) | 3.83 (2.74-4.92) |  |  |  |  |

*Note: * p-value < 0.05 with Bonferroni correction for differences among periods: 1- Pre-First lockdown Vs First lockdown; 2- Post-first lockdown Vs first lockdown, 3- Second lockdown Vs Post-first lockdown, 4 - Post-second lockdown Vs second lockdown*

**Table 2. Weekly count and incidence of Emergency Department access/Hospitalization due to psychiatric conditions among young individuals.**

|  | Periods |  |  |  |  | Relative Change (%) |  |  |  |
| --- | --- | --- | --- | --- | --- | --- | --- | --- | --- |
| Outcome | **Pre-first lockdown** | **First lockdown** | **Post-first lockdown** | **Second lockdown** | **Post-second lockdown** | **First lockdown Vs Pre-first lockdown** | **Post-first lockdown Vs first lockdown** | **Second lockdown Vs Post-first lockdown** | **Post-second lockdown Vs second lockdown** |
| Weekly count | |  |  |  |  |  |  |  |  |
| Mean ± SD | 20.92 ± 5.75 | 7.69 ± 4.76 | 15.24 ± 4.41 | 16.15 ± 5.34 | 17.68 ± 6.16 | -63.25 * | 98.22 * | 5.99 | 9.49 |
| Median (Q1-Q3) | 21 (17-25) | 7 (4.75-10.25) | 15 (11-19) | 15 (12-19) | 18 (13-21) |  |  |  |  |
| Weekly incidence per 100,000 | | | | | | | | | |
| Mean ± SD | 10.67 ± 2.95 | 4.05 ± 2.51 | 8.03 ± 2.33 | 8.64 ± 2.87 | 9.61 ± 3.32 | -62.01 * | 98.22 * | 7.61 | 11,16 |
| Median (Q1-Q3) | 10.69 (8.62-12.89) | 3.69 (2.5-5.4) | 7.91 (5.8-10.02) | 8.06 (6.45-10.21) | 9.85 (7.05-11.39) | |  |  |  |

*Note: * p-value < 0.05 with Bonferroni correction for differences among periods: 1- Pre-First lockdown Vs First lockdown; 2- Post-first lockdown Vs first lockdown, 3- Second lockdown Vs Post-first lockdown, 4 - Post-second lockdown Vs second lockdown*
